# An open chat with… Koji Yamanaka

**DOI:** 10.1002/2211-5463.13763

**Published:** 2024-01-12

**Authors:** Ioannis Tsagakis, Koji Yamanaka

**Affiliations:** ^1^ *FEBS Open Bio* Editorial Office Cambridge UK; ^2^ Department of Neuroscience and Pathobiology, Research Institute of Environmental Medicine Nagoya University Japan; ^3^ Department of Neuroscience and Pathobiology Nagoya University Graduate School of Medicine Japan; ^4^ Institute for Glyco‐core Research Nagoya University Japan; ^5^ Center for One Medicine Innovative Translational Research (COMIT) Nagoya University Nagoya Japan

## Abstract

Koji Yamanaka is a Professor at the Research Institute of Environmental Medicine at Nagoya University of Japan. His research interests lie in understanding the mechanism of onset and progression of motor neuron disease as well as the role of glial cells in Alzheimer's disease neuroinflammation. Koji has been serving on the *FEBS Open Bio* Editorial Board since 2013. In this interview, he explains the implications of recent findings in neurobiology for amyotrophic lateral sclerosis, provides updates on the research environment in Japan and discusses how editors might use their position to positively influence academic culture.

## Graphical abstract

Koji Yamanaka is a professor at the Research Institute of Environmental Medicine of Nagoya University and a member of the *FEBS Open Bio* Editorial Board. Here, he explains the implications of recent findings in neurobiology for amyotrophic lateral sclerosis, provides updates on the research environment in Japan and discusses how editors might use their position to positively influence academic culture.

## What inspired you to pursue a career as a researcher?

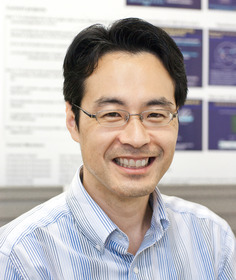

I started my career as a clinical neurologist. During my residency in Neurology at Kyoto University in Japan, I had experience working with many patients with neurological diseases, for whom limited therapies are available. This motivated me to become engaged in research on neurodegenerative diseases.

## Please tell us about your research

I have been focusing on amyotrophic lateral sclerosis (ALS), one of the intractable neurodegenerative diseases affecting motor neurons. Our team has identified that multiple cell types, such as neurons and glial cells, are actively participating in disease onset and progression. In contrast to previous research mainly focusing on affected neurons in ALS, our strategy aims to elucidate the crosstalk between motor neurons and glial cells and how that links to ALS initiation and progression which may provide clues to treat this disease.

## What is your favourite topic in neuroscience?

My favourite topic is understand the role of neuroinflammation and proteostasis in neurodegenerative diseases, such as ALS and Alzheimer's disease (AD). Recent genome‐wide association studies in AD identified many risk genes linked to microglia, the innate immune cells of the brain. This motivated us to extend our research to the understanding of AD.

## Could you tell us a bit about the AD ‘In the Limelight’ Issue?

Significant advances have been made in AD research, such as understanding the role of glial cells and neuroinflammation, amyloid and tau imaging for diagnosis, and the antiamyloid antibody for therapy. For this ‘In the Limelight’ issue, we invited four review articles by experts in AD. Sahara and Higuchi reviewed recent advances in tau biology, *in vivo* diagnostic imaging and the development of tau‐targeted therapies [1]. Kimura and Tomita provided a review focusing on the structural diversity of tauopathy and its implication in disease and biomarkers [2]. Kawade and Yamanaka reviewed brain lipid metabolism in AD and its implication in oligodendrocyte abnormalities, an under‐investigated aspect of AD [3]. Finally, Dadwal and Heneka focused on microglia, innate immune cells in the brain and their heterogeneity in AD [4]. I hope that this ‘In the Limelight’ issue will attract the keen interest of *FEBS Open Bio* readers and encourage more neuroscience manuscripts to be published in this journal.

## Can you elaborate on why glial cell function is so important for motor neurons that you think a cure for ALS may come from harnessing the biology of these cells?

Our previous studies using inherited ALS model mice expressing an ALS‐linked mutant form of superoxide dismutase 1 (SOD1) revealed that the damage within microglia or astrocytes accelerated disease progression in mice. Whereas previous studies mainly focused on the pathogenic mechanism of motor neurons, our results shed light on the active role of glial cells in the disease progression of ALS [5, 6].

## There are a lot of groups that are working on gene therapy as a cure for ALS and spinal muscular atrophy (SMA). What are some challenges or opportunities with these approaches and could glial cell function play a role in facilitating their success?

Recent developments in nucleic acid medicine substantially altered the disease course and outcome of SMA patients, especially by restoring splicing of the *SMN* gene. For SOD1‐linked ALS, antisense oligonucleotides aimed at eliminating SOD1 confer neuroprotection in patients. I feel that those drugs are mainly aimed at motor neurons, but perhaps there are some additional effects on glial cells.

## You made a very early move from medicine to biology when you moved from a medical residency in 1992 to a PhD in 1996. Was biology popular at the time in Japan and what led to your move?

In Japan, many young medical doctors are encouraged to enter the graduate school of medicine after a few years of an initial clinical career. At that time, I was really motivated to learn basic biochemistry and molecular biology which are crucial to conduct basic research on neurological disease. Personally, I felt I really made the move into basic medical/ biological research when I moved to the University of California, San Diego as a postdoctoral fellow.

## You spent most of your training years in Japan but then moved to the United States in 2001. Did you ever face any challenges being a Japanese researcher working abroad?

In the laboratory, I worked with many postdocs and graduate students who originated from various countries and enjoyed such a mixed cultural atmosphere. I felt that it is important for our life and science to experience and understand such diversity.

## Could you tell us what you have found to be unique in Japan throughout your career about being a researcher there?

I am concerned that there is a decrease in the number of individuals who are entering PhD courses and pursuing academic careers in science in Japan. One of the reasons is a relatively limited career path for PhD scholars in Japan. Industries prefer to hire undergraduates rather than PhD scholars. I really hope that the employment practices of industries will change to increase career options from the PhD route.

## How has the academic work environment changed in Japan over the last 20 years? Are there more initiatives for mobility (allowing researchers to move across institutes) and tenure than in the past?

We face a decline in research output and impact in Japan that has been taking place over the last 20 years [7]. One of the reasons is a reduction in the intramural research budget for universities. The government shifted funding to universities into competitive grants. In addition, there is a lack of supporting personnel, who take care of the numerous paperwork and laboratory management. Therefore, the time that researchers can spend on research has decreased in the last 20 years. The government has acknowledged this reality and has attempted to counteract it. I hope it is not too late to attract young students into academia.

## What is your opinion on gender balance in the academic environment of biological sciences in Japan?

The gender balance is an important issue in academia as well as other fields. The gender balance is still insufficient in the biological science field. Nagoya University has taken an initiative to participate in HeForShe (a global initiative to promote gender diversity led by United Nations Women) for years. I think such organizational initiatives are important to increase the number of women scientists and provide a sustainable work environment.

## Do you feel being a member of the *FEBS Open Bio* Editorial Board allows you to better engage with the neuroscience community?

I really hope that *FEBS Open Bio* can offer the neuroscience community an opportunity to publish their latest scientific findings. As an open‐access journal (with a competitive APC price), the works published in *FEBS Open Bio* will be quickly and easily recognized by researchers worldwide. I wish that *FEBS Open Bio* could accept rapid communication articles to publish competitive works with other scientists. As a president of the Japan Neuroscience Society, I also would like to promote and introduce neuroscience through editorial work at *FEBS Open Bio*.

## Does being a member of several journal editorial boards enable you to positively influence the academic culture/environment?

As an editorial board member from Japan, I put my efforts into increasing the visibility of Asian scientists through editorial work. Most of the large publishers have originated (or are located) in the United States or Europe, and thus, I am concerned that Asia and other regions of the world are underrepresented in science.

## What is the most memorable thing that has happened to you in the laboratory?

I was very fortunate to become a principal investigator at the age of 38 and to have my own laboratory at the RIKEN Brain Science Institute, Japan, in 2006. In Japan, there are very few opportunities for young researchers to become a PI. I would say that the most memorable event for me was celebrating the anniversary of my laboratory with members of the group.
